# Angiotensinergic and GABAergic transmission in the medial preoptic area: role in urinary bladder and cardiovascular control in female rats

**DOI:** 10.3389/fphys.2023.1224505

**Published:** 2023-09-13

**Authors:** Sergio A. Daiuto, Rodrigo P. de Carvalho, Bárbara do Vale, Nuha A. Dsouki, Gisele Giannocco, Eduardo M. Cafarchio, Patrik Aronsson, Monica A. Sato

**Affiliations:** ^1^ Department Morphology and Physiology, Faculdade de Medicina do ABC, Centro Universitario FMABC, Santo Andre, Brazil; ^2^ Department Medicine, Federal University of Sao Paulo, Sao Paulo, Brazil; ^3^ Department Pharmacology, Institute of Neuroscience and Physiology, Sahlgrenska Academy, University of Gothenburg, Gothenburg, Sweden

**Keywords:** medial preoptic area, angiotensin II, GABA, AT-1 receptors, ACE, arterial pressure, heart rate, micturition

## Abstract

**Introduction:** The medial preoptic area (mPOA) participates in thermoregulatory control and blood pressure modulation as shown by studies with electrical stimulation of this area or cobalt chloride injection, a non-selective synapse inhibitor. This study aimed to investigate whether angiotensin II (Ang II) and GABA could act or not in the mPOA to mediate the cardiovascular and micturition control pathways.

**Methods:** Female Wistar rats were submitted to stereotaxic surgery for implantation of a guide cannula into the mPOA 7 days prior to the experiments. Afterwards, the animals were isoflurane- anesthetized and submitted to the catheterization of the femoral artery and vein and urinary bladder cannulation for mean arterial pressure (MAP), heart rate (HR), and intravesical pressure (IP) recordings, respectively. After the baseline MAP, HR, and IP recordings for 15 min, Ang II (0.1 nM, 1 μL), losartan (AT-1 receptor antagonist, 100 nM, 1 μL), GABA (50 mM, 1 μL) or saline (1 μL) were injected into the mPOA, and the variables were measured for additional 30 min. In a different group of rats, the AT-1 receptor, angiotensin II converting enzyme (ACE), and GABAa receptor gene expression was evaluated in mPOA samples by qPCR. The data are as mean ± SEM and submitted to One-way ANOVA (Tukey posttest) or paired Student t-test (P <0.05).

**Results:** The injection of Ang II into the mPOA evoked a significant hypotension (−37±10 mmHg, n = 6, *p* = 0.024) and bradycardia (−47 ± 20 bpm, *p* = 0.030) compared to saline (+1 ± 1 mmHg and +6 ± 2 bpm, n = 6). A significant increase in IP was observed after Ang II injection into the mPOA (+72.25 ± 17.91%, *p* = 0.015 vs. −1.80 ± 2.98%, n = 6, saline). No significant changes were observed in MAP, HR and IP after the losartan injection in the mPOA compared to saline injection. Injection of GABA into the mPOA evoked a significant fall in MAP and HR (−68 ± 2 mmHg, n = 6, *p* < 0.0001 and −115 ± 14 bpm, n = 6, *p* = 0.0002 vs. −1 ± 1 mmHg and +4 ± 2 bpm, n = 6, saline), but no significant changes were observed in IP. The AT-1 receptor, ACE and GABAa receptor mRNA expression was observed in all mPOA samples.

**Discussion:** Therefore, in female rats, Ang II mediated transmission in the mPOA is involved in the cardiovascular regulation and in the control of central micturition pathways. A phasic control dependent on AT-1 receptors in the mPOA seems to be involved in the regulation of those cardiovascular and intravesical 3 parameters. In contrast, GABAergic transmission in the mPOA participates in the pathways of cardiovascular control in anesthetized female rats, nevertheless, this neurotransmission is not involved in the micturition control.

## 1 Introduction

The preoptic area is long known for its involvement in the modulation of the autonomic nervous system, demonstrated for instance by changes in blood pressure after electrical stimulation of this area in anesthetized cats (Kabat et al., 1935). The bradycardia evoked by electrical stimulation of the medial preoptic area (mPOA) is significantly attenuated by vagotomy, suggesting that this region exerts vagal activation and sympathetic inhibition (Wang and Ranson, 1941). Nevertheless, Folkow et al. (1959) have demonstrated a bradycardia and pronounced drop in blood pressure after electrical stimulation of the preoptic area in vagotomized cats with bilateral carotid occlusion, evidencing a possible sympathoinhibitory function. Despite these earlier findings, the effects on blood pressure and heart rate induced by electrical stimulation of the mPOA could not be explicitly attributed to this area. Electrical stimulation activates both neurons in the preoptic area as well as other passing fibers, which can lead to activation of neurons located outside the preoptic area.

The mPOA participates in thermoregulatory control and promotes blood pressure modulation, evidenced by studies using injection of cobalt chloride, a non-selective inhibitor of synapses (Fassini et al., 2017). It also presents neuron cell bodies with immunohistochemical labeling for angiotensin II (Ang II) (Lind et al., 1985). The mPOA contains mRNA for angiotensinogen and low density of fibers with immunoreactivity for Ang II, modest immunoreactivity for AT-1 receptors and moderate amounts of angiotensin-converting enzyme (ACE) (Bunnemann et al., 1993). However, immunoreactivity for angiotensinogen has not been reported.

Impairment of urinary bladder functions, such as difficulties in urine storage and bladder emptying affects men and women, as well as children worldwide. Among the urinary bladder dysfunctions, urinary incontinence has been reported with higher prevalence in women (Aoki et al., 2017). The central control of micturition involves a complex mechanism, which is still not fully understood. Nevertheless, maintenance of urinary excretion and storage depends on reflex mechanisms, with the initiation of voiding influenced by the Pontine Micturition Center (PMC). In contrast, urine storage is modulated by the Pontine Urine Storage Center (PUSC), located ventrolaterally to the PMC (De Groat, 1998). In rats, the PMC corresponds to Barrington’s nucleus and acts as a modulator of the micturition reflex. The mechanism affects bladder pressure and volumetric control, as well as interferes with the coordination of the actions of the detrusor muscle and the urethral sphincter (Sugaya et al., 2005).

Sugaya et al. (1997) demonstrated that, in spite of the micturition in neonatal rats does not depend on neural mechanisms in the brain, many neurons in various brain regions are labeled by the pseudorabies virus (PRV) injected into the urinary bladder of rat pups at 2 and 10 days of age. This indicates that the bladder and brain are connected at very early age. However, the distribution of PRV-infected neurons is somewhat broader than in adult mice. In the first 72 h after PRV administration, the labeling is more prominent in PMC. Other neuronal populations are labeled at slightly longer times (78–84 h), including the raphe magnus nucleus, A5 and A7 clusters, parapyramidal reticular formation, periaqueductal gray (PAG), locus coeruleus, hypothalamus lateral, mPOA and frontal cortex. These areas are also labeled in adult animals. In addition, many of those areas are correspondent to sites where electrical stimulation facilitates or inhibits bladder activity in adult animals (De Groat et al., 2015).

Muscimol, a GABAa receptor agonist, injections in the mPOA increases body temperature, blood pressure and heart rate in conscious freely moving rats by affecting different circuitries in this area, i.e., one involving orexin neurons, and a separate orexin-independent circuit activated by prostaglandin E2 (Rusyniak et al., 2011). Nevertheless, it is unknown whether the GABAergic pathway could be also involved in the micturition control pathways.

Although the mPOA is one of the areas in the brain which is retrogradely labeled by PRV injected in the urinary bladder, the neurotransmission and the role of mPOA in the urinary bladder control is still largely unknown. Bastos et al. (1994) have shown that Ang II elicits dipsogenic effect and pressor response in conscious rats when injected in the mPOA. However, it is not clear if Ang II in the mPOA participates in the micturition control pathways. Therefore, in this study we focused to investigate whether Ang II and GABA could act in the pathways of mPOA to mediate intravesical pressure and/or cardiovascular control in female anesthetized rats. We also evaluated the gene expression of 1) ACE and AT-1 receptors in the mPOA in order to understand if Ang II is locally synthetized; 2) GABAa receptors to demonstrate the existence of these receptors in the mPOA neurons, where the neurotransmitter GABA could bind to exert its effects.

## 2 Materials and methods

### 2.1 Animals

Adult female Wistar rats (∼260 g), provided by the Animals Care of Centro Universitario FMABC, were used. The animals had access to standard chow pellets (Nuvilab®) and tap water *ad libitum*. Before the stereotaxic surgery, rats were maintained in plastic cages in groups of 4 animals, and after the surgical procedure, each rat was placed in an individual plastic cage. The light-dark cycle of the Animal Care in the Physiology laboratory at Centro Universitario FMABC was set as 12 h each. The humidity was also controlled at ∼70%, and the room temperature was maintained at approximately 23°C. All procedures were performed in accordance with the National Institutes of Health (NIH) Guide for the Care and Use of Laboratory Animals, and were approved by the Animal Ethics Committee of the Faculdade de Medicina do ABC/Centro Universitario ABC (protocol number 02/2021).

### 2.2 Implantation of guide cannulas in the medial preoptic area

Rats were initially sedated with 2% isoflurane in 100% O_2_ and then anesthetized with ketamine (50 mg/kg, i.p.) and xylazine (10 mg/kg, i.m.). Afterwards, the animals were placed in a stereotaxic apparatus (David Kopf®). Antisepsis in the surgical field was performed using polyvinyl-pyrrolidone (PVPI). The cranial surface was exposed to visualize the sutures (bregma and lambda). The animal’s head was horizontally aligned based on the dorsoventral parameters measured at the level of the bregma and lambda, which should be coincidental. Two jeweler screws were placed in the animal’s skull in order to allow the guide cannula to be anchored to the screws with acrylic cement. A hole was made in the skullcap with the aid of a dental bur and a stainless-steel guide cannula with 12 mm length (23 gauge, 0.642-mm OD, 0.337-mm ID, BD, Juiz de Fora, Brazil) was inserted and positioned towards the mPOA. The parameters to achieve the mPOA were measured as follows: 0.0 mm from bregma, ±0.7 mm lateral from midline, and −7.7 mm ventral from the cranial surface at the anteroposterior level on the spot for insertion of the guide cannula. The screws, the skullcap’s hole, and the skull’s surface were covered with self-curing dental acrylic cement (Jet Líquido Clássico®). At the end of the surgery, Veterinary Pentabiotic for Small Animals (2,000 U/mL, 0.1 mL/rat, i.m., Fort Dodge Saude Animal, Campinas, Brazil) was administered in a single dose as a prophylactic measure, as well as meloxicam (1.0 mg/kg/day, S.C., per day, Maxicam, Ourofino Saude Animal, Campinas, Brazil) for 3 days.

### 2.3 Catheterization of the femoral artery and vein

Rats anesthetized with 2% isoflurane in 100% O_2_ were submitted to the cannulation of the femoral artery and vein through the insertion of a polyethylene tube (PE-50 connected to PE-10, Clay Adams, NJ, United States) for pulsatile arterial pressure (PAP), mean arterial pressure (MAP) and heart rate (HR) recordings in the data acquisition system (PowerLab 16 SP, ADInstruments, Castle Hill, AU), as well as drug administration, respectively.

### 2.4 Bladder cannulation and intravesical pressure measurement

Rats anesthetized with 2% isoflurane in 100% O_2_ were submitted to urinary bladder cannulation by inserting a polyethylene tube (PE-50 connected to PE-10, Clay Adams, NJ), which was connected to a pressure transducer for intravesical pressure (IP) recording in the data acquisition system (PowerLab 16 SP, ADInstruments, Castle Hill, AU).

### 2.5 Drug microinjection in the mPOA

Drug microinjections in the mPOA were performed using a needle (27 gauge, 0.413-mm O.D., 0.210-mm I.D., 13-mm length, Injex, São Paulo, Brazil) connected to a 10-μL Hamilton syringe (Reno, NV, United States) by polyethylene tubing (PE-10, Clay Adams, NJ, United States). The volume of all the drugs injected into the mPOA was 1 μL, as previously reported in the studies by Bastos et al. (1994).

### 2.6 Histological analysis

**FIGURE 1:**
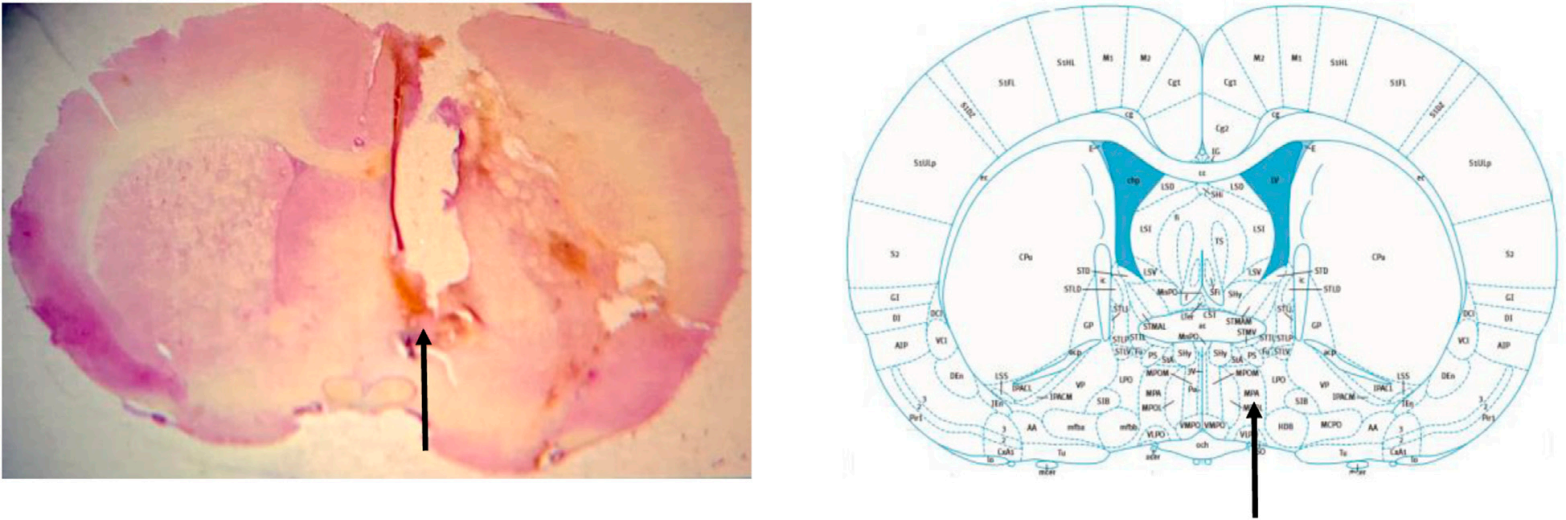
Photomicrograph of a rat from the experimental group demonstrating the drug injection site in the medial preoptic area (arrow) (left image). Schematic representation of the location of the medial preoptic area (arrow) in a section of the brain, according to the atlas of Paxinos and Watson. (2009).

At the end of the experiments, the animals were deeply anesthetized with i.v. injection of sodium thiopental 100 mg/kg (Cristália, Itapira, SP, Brazil) and 4% Chicago Sky Blue dye (Sigma Aldrich, St. Louis, MO, United States) was administered in a volume of 1 µL in the mPOA in order to determine the site of drug injections. Then, the rib cage was opened to expose the heart, and roughly 40 mL of formalin solution (10%) (Synth, Diadema, Brazil) was perfused intracardially. Afterwards, the brain was harvested and kept in the same formalin solution for at least 48 h. Subsequently, the brain was sectioned in a freezing microtome (Leica Biosystems, Buffalo Grove, IL, United States). The histological sections (40 µm) were stained with hematoxylin-eosin and analyzed in a light field microscopy (Nikon, Eclipse E-200, Tokyo, Japan). Only animals with histological confirmation of microinjection sites in the mPOA were considered in this study (Figure 1).

### 2.7 Gene expression of AT-1 receptors, ACE and GABAa receptor in the mPOA by quantitative real-time polymerase chain reaction (qPCR)

Total RNA was isolated from frozen mPOA samples with TRIzol Reagent® (Thermo Fisher Scientific) according to the manufacturer’s protocol. RNA integrity was checked by agarose gel electrophoresis, and the RNA purity reached the following criteria: A260/280 ≥ 1.8. The extracted total RNA concentration was measured using a NanoDropTM (One-One c) spectrophotometer (Thermo Fisher Scientific), and 1 μg of total RNA was subjected to reverse transcription reaction. Complementary DNA (cDNA) synthesis was generated using ImPromIITM Reverse Transcription System (Promega, Madison, WC, United States) according to the manufacturer’s protocol. Quantitative real-time PCR (qPCR) was carried out using 2 μL of cDNA and the Eva GreenTM qPCR Mix Plus (Solis BioDyne, Tartu, Estonia) in the ABI Prism 7,500 Sequence Detection System (Applied Biosystems, Foster City, CA) to amplify specific primers sequences for AT-1 receptor, ACE, GABAa receptor, Cyclophilin A (housekeeping gene), GAPDH (housekeeping gene), 18S rRNA (housekeeping gene). The procedure consisted of an initial step of 10 min at 95°C, followed by 45 cycles of 20 s each at 95°C, 20 s at 58°C, and 20 s at 72°C. Gene expression was determined by cycle threshold (CT), and all values were expressed, using cyclophilin A or GAPDH or 18S rRNA as an internal control.

The forward and reverse primers sequences (Thermo Fisher Scientific) for rats used in this study follow below:

AT-1 receptor:

(forward)—5-AGTCCTGTTCCACCCGATCA-3´

(reverse)—5´-TCCAGACAAAATGCCAGCCA—3´

ACE:

(forward)—5´-CGGTTTTCATGAGGCTATTGG-3´

(reverse)—5´-TCGTAGCCACTGCCCTCACT-3´

GABAa receptor:

(forward)—5’-GAGCACGCAGAGTCCATGA-3’

(reverse)—5’-GAGAGGATCGCGGTGAGC-3’

Cyclophilin A (housekeeping gene):

(forward)—5´-CCCACCGTGTTCTTCGACAT-3´

(reverse)—5´-CTGTCTTTGGAACTTTGTCTGCAA-3´

GADH (housekeeping gene):

(forward)—5´-ACCACAGTCCATGCCATCAC -3´

(reverse)—5´-TCCACCACCCTGTTGCTGTA -3´

18S rRNA

(forward)—5´-CATTCGAACGTCTGCCCTAT -3´

(reverse)—5´-GTTTCTCAGGCTCCCTCTCC -3´

## 3 Experimental protocol

### 3.1 Effect of angiotensin II injection in the mPOA on intravesical pressure and cardiovascular parameters (N = 6)

This experimental protocol was aimed to assess whether or not the injection of Angiotensin II in the mPOA could change the intravesical pressure and if these changes would be related to changes in arterial pressure.

**FIGURE 2:**
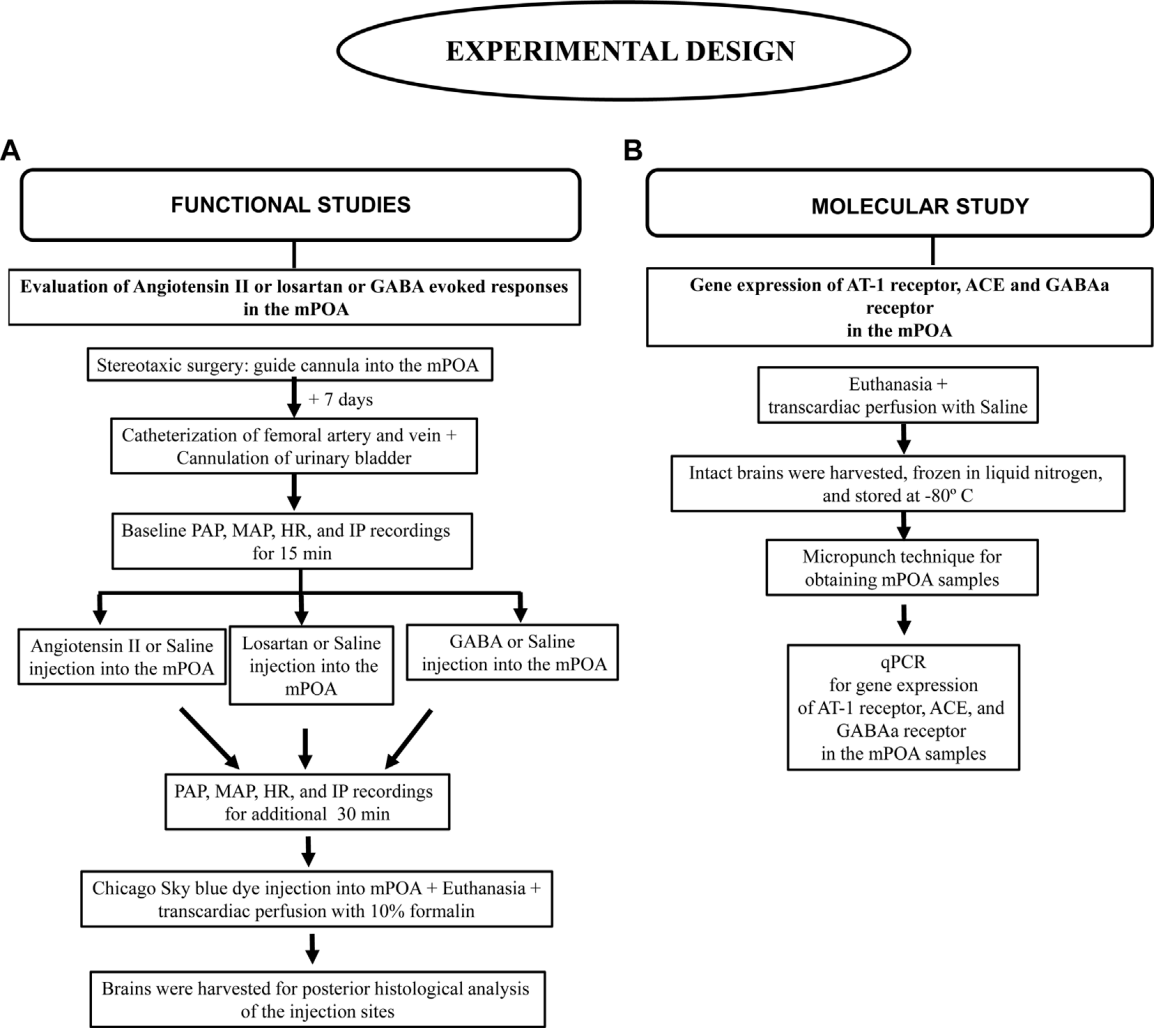
Flowchart depicting the experimental design details. **(A)** Functional studies, **(B)** Molecular study.

The animals were initially submitted to the stereotaxic surgery to implant a guide cannula in the mPOA. After 7 days, the animals were anesthetized with 2% isoflurane in 100% O_2_ for catheterization of the femoral artery and vein and urinary bladder cannulation. The animals were maintained under the same anesthesia over the whole experimental procedure. After baseline recording of PAP, MAP, HR, and IP for 15 min, Angiotensin II (0.1 nM/μL, 1 µL) or saline (1 µL) was injected into the mPOA, and the variables were measured for additional 30 min (Figure 2).

### 3.2 Effect of losartan (AT-1 receptor antagonist) injection in the mPOA on intravesical pressure and cardiovascular parameters (N = 6)

This experimental protocol was intended to assess whether or not the blockade of AT-1 receptors with losartan injection in the mPOA could change the intravesical pressure and if these changes would be related to changes in arterial pressure.

In a different group of rats from section 8.1., the same surgical procedures were performed as described above (section 8.1). After measuring the baseline PAP, MAP, HR, and IP for 15 min, losartan (100 nM/μL, 1 µL) or saline (1 µL) was injected into the mPOA, and the variables were recorded for additional 30 min (Figure 2).

### 3.3 Effect of GABA injection in the mPOA on intravesical pressure and cardiovascular parameters (N = 6)

This experimental protocol was aimed to evaluated whether or not the GABAergic inhibition of mPOA could change the intravesical pressure and if these changes would be related to changes in arterial pressure. In addition, this protocol intended to investigate if the possible changes in intravesical pressure and cardiovascular parameters showed any similarity to the responses elicited by activation or blockade of AT-1 receptors.

In a different group of rats from that used in sections 8.1 and 8.2, the same surgical procedures reported above (section 8.1) were carried out. After baseline measurement of PAP, MAP, HR and IP for 15 min, GABA (50 mM/μL, 1 µL) or saline (1 µL) was injected into the mPOA, and the variables were recorded for additionally 30 min (Figure 2).

### 3.4 Gene expression of AT-1 receptor, ACE, and GABAa receptor in the mPOA by quantitative real-time polymerase chain reaction (qPCR)

This experimental protocol was intended to evaluate if the genes for AT-1 receptor, ACE, and GABAa receptor were expressed in the mPOA of rats.

A different group of rats from that used in sections 8.1, 8.2 and 8.3 was used. Animals were deeply anesthetized with isoflurane 4% in 100% O_2_ and submitted to a thoracotomy for transcardiac perfusion of 40 mL of phosphate buffered saline. After that, a craniotomy was performed and the brain was harvested, immediately frozen in liquid nitrogen, and stored at −80°C in an ultrafreezer (Thermo Fisher Scientific®) until the day of total RNA extraction with the TRizol® reagent. To obtain mPOA samples, the brain was sliced and a micropunch was performed on frozen sections of the rat brain. The further procedures for gene expression of AT-1 receptor, ACE, GABAa receptor by qPCR were performed as described in section 7 (Figure 2).

## 4 Statistical analysis

A Shapiro-Wilk test for normality was used for verifying the data distribution. Once the results fit to a normal distribution, they were expressed as mean ± S.E.M., and subjected to the One-way ANOVA followed by Tukey posttest to compare the MAP, HR and IP responses evoked by angiotensin II, losartan and saline injections in the mPOA. Paired Student´s t-tests were used to compare the MAP, HR, and IP responses induced by GABA injection in the mPOA. Statistical analysis was performed using Graph Pad Prism 9.5.0. The significance level was set at *p* <0.05.

## 5 Results

### 5.1 Responses on intravesical pressure and cardiovascular parameters in anesthetized rats evoked by angiotensin II or losartan injection in the mPOA

At baseline (before the injections in the mPOA), the SAP, DAP, MAP, and HR was 131 ± 4 mmHg, 99 ± 4 mmHg, 110 ± 3 mmHg and 433 ± 23 bpm, respectively (Ang II group, n = 6), 124 ± 3 mmHg, 106 ± 2 mmHg, 112 ± 2 mmHg and 448 ± 17 bpm, respectively (losartan group, n = 6), and 120 ± 2 mmHg, 105 ± 3 mmHg, 110 ± 3 mmHg and 424 ± 11 bpm, respectively (saline group, n = 6). The baseline IP (before the injections in the mPOA) was 7.08 ± 0.80 mmHg (Ang II group), 8.17 ± 0.65 mmHg (losartan group) and 6.99 ± 0.98 mmHg (saline group).

**FIGURE 3:**
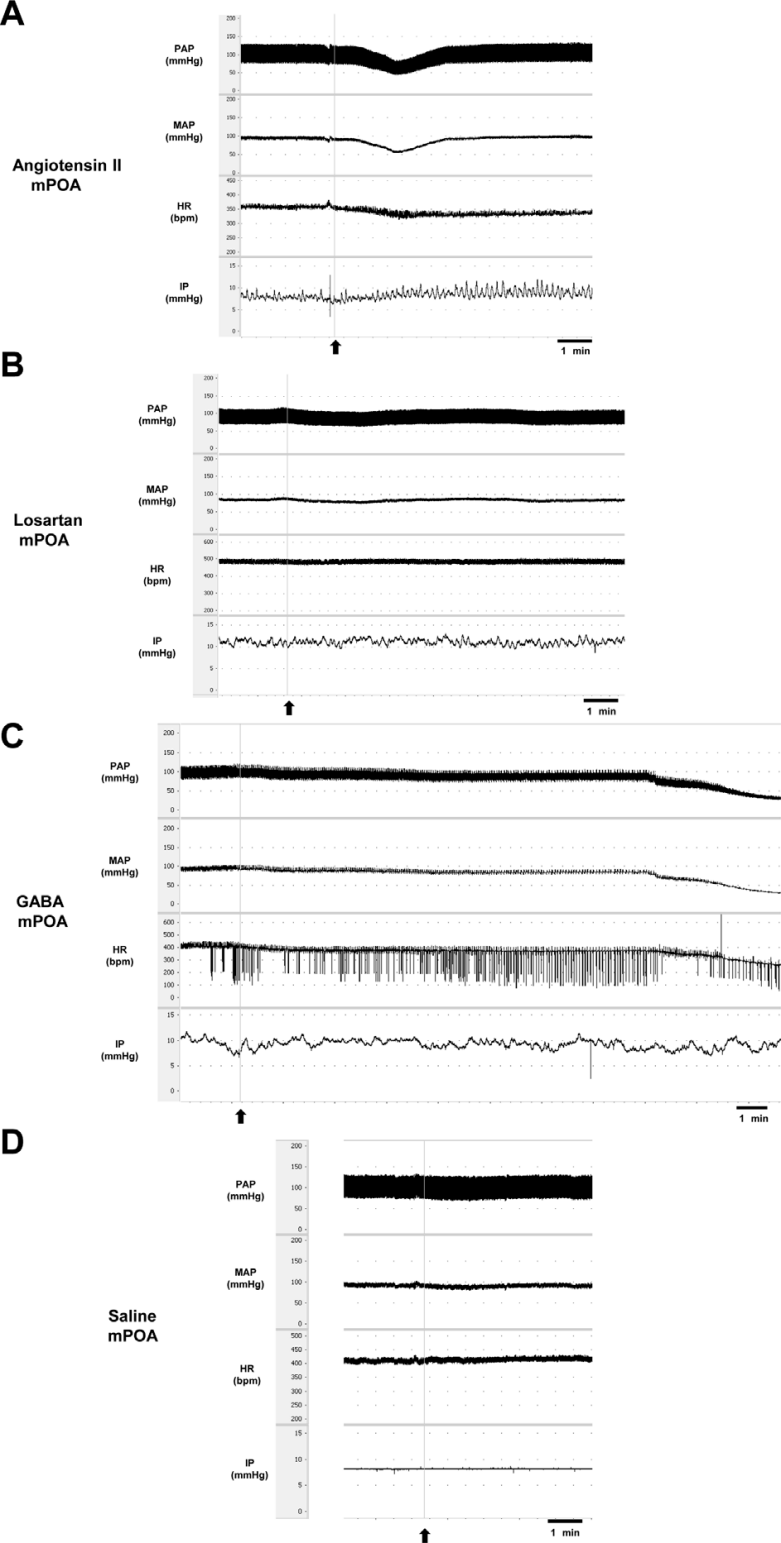
Tracings showing the baseline pulsatile arterial pressure (PAP, mmHg), mean arterial pressure (MAP, mmHg), heart rate (HR, bpm), intravesical pressure (IP, mmHg), and the responses induced by **(A)** angiotensin II (0.1 nM/μL, 1 µL) or **(B)** losartan (100 nM/μL, 1 µL) or **(C)** GABA (50 mM, 1 µL) or **(D)** saline (1 µL) into the medial preoptic area. Arrows indicate the moment of injections in the medial preoptic area.

**FIGURE 4:**
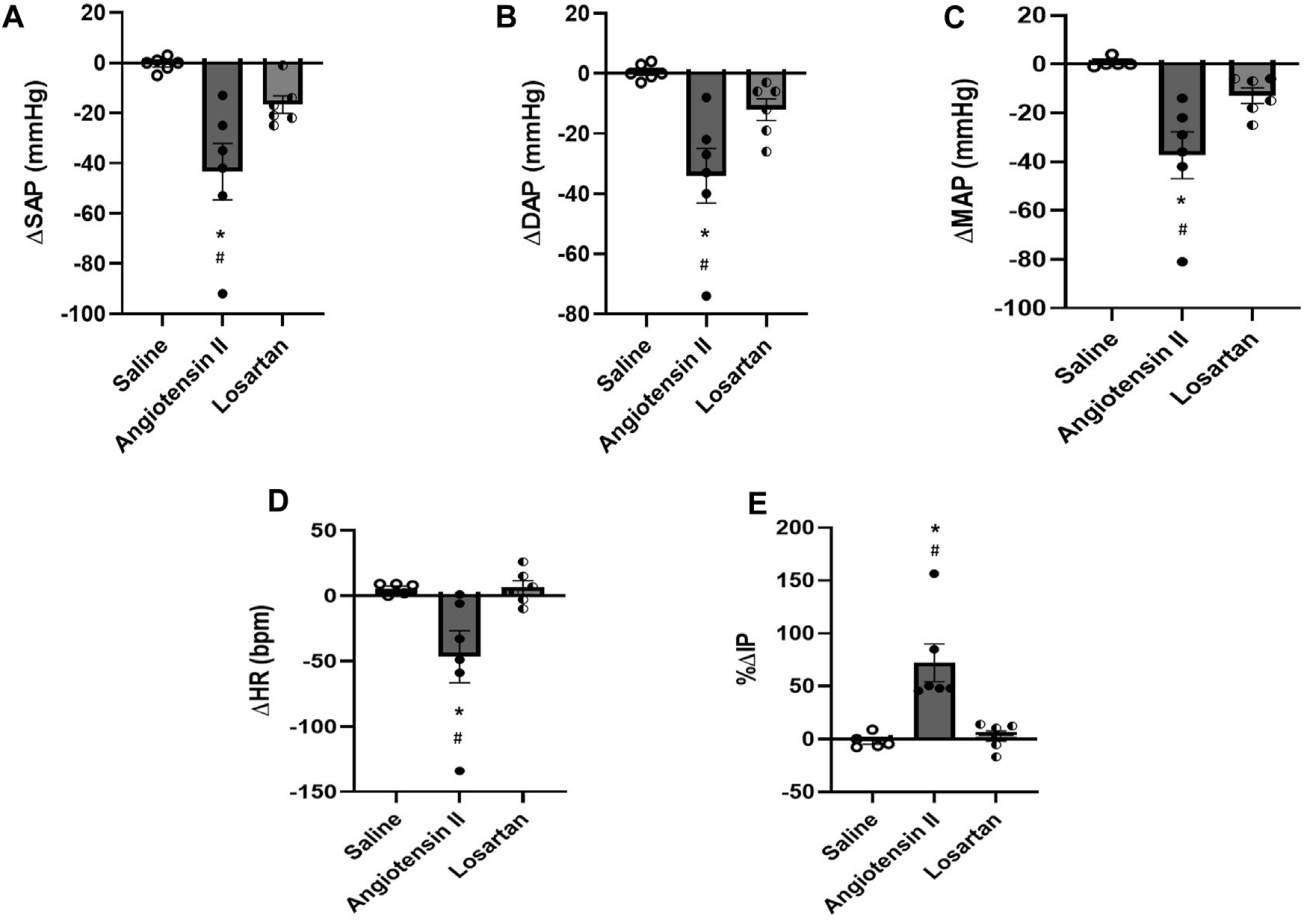
**(A)** Change in systolic arterial pressure (ΔSAP, mmHg), **(B)** diastolic arterial pressure (ΔDAP, mmHg), **(C)** mean arterial pressure (ΔMAP, mmHg), **(D)** change in heart rate (ΔHR, bpm), and **(E)** percent change in intravesical pressure (%ΔIP) evoked by injection of saline (1 µL), angiotensin II (0.1 nM/μL, 1 µL) or losartan (100 nM/μL, 1 µL) into the medial preoptic area (n = 6). *p < 0.05 vs. saline (One-way ANOVA, followed by Tukey posttest), #*p* <0.05 vs. losartan (One-way ANOVA, followed by Tukey posttest).

Injection of Ang II (n = 6) into the mPOA promoted a significant reduction in SAP (−43 ± 11 mmHg, *p* = 0.003), DAP (−34 ± 9 mmHg, n = 0.004), MAP (−37 ± 10 mmHg, *p* = 0.003) and HR (−47 ± 20 bpm, *p* = 0.030) compared to saline injection (−1 ± 1 mmHg, 1 ± 1 mmHg, +1 ± 1 mmHg and +6 ± 2 bpm, respectively, n = 6). In contrast, we observed a significant increase in IP (+72.25 ± 17.91%, *p* = 0.015) after Ang II injection into the mPOA compared to saline injection (−1.80% ± 2.98%) (Figures 3A,D; Figure 4). The latency for the peak response in MAP and HR evoked by Ang II in the mPOA was roughly 4 min, whereas the peak response in IP was achieved at ∼6 min after Ang II injection in the mPOA. The duration of the Ang II evoked-response was different on the cardiovascular parameters compared to IP, as the reduction in MAP and HR lasted between 7–10 min, whereas the increase in IP persisted between 10–14 min.

Blockade of AT-1 receptors with losartan injection (n = 6) in the mPOA elicited no significant changes in SAP (−15 ± 5 mmHg, *p* = 0.306), DAP (−12 ± 4 mmHg, *p* = 0.350), MAP (−13 ± 3 mmHg, *p* = 0.399) and HR (+6 ± 5 bpm, *p* = 0.999) compared to saline injection (−1 ± 1 mmHg, 1 ± 1 mmHg, +1 ± 1 mmHg, and +6 ± 2 bpm, respectively, n = 6). Similarly, no significant changes in IP were observed after losartan injection (+2.92 ± 4.93%, n = 6, *p* = 0.957) compared to saline (−1.80% ± 2.98%) (Figures 3B,D; Figure 4). The latency for the peak in the weak changes in MAP and HR was observed at 9 min after losartan injection into the mPOA, whereas the small peak change for IP was achieved at 12 min after losartan injection into the mPOA. The weak change in MAP, HR and IP elicited by losartan injection in the mPOA lasted between 14–17 min.

### 5.2 Responses on intravesical pressure and cardiovascular parameters elicited by GABA injection in the mPOA

At baseline (before the injections in the mPOA), the SAP, DAP, MAP, and HR was 119 ± 5 mmHg, 97 ± 3 mmHg, 104 ± 3 mmHg and 415 ± 12 bpm (GABA group, n = 6) and 117 ± 5 mmHg, 94 ± 2 mmHg, 102 ± 3 mmHg and 407 ± 12 bpm, respectively (saline group, n = 6). The baseline IP was 9.00 ± 0.82 mmHg (GABA group), and 8.41 ± 0.74 mmHg (saline group).

**FIGURE 5:**
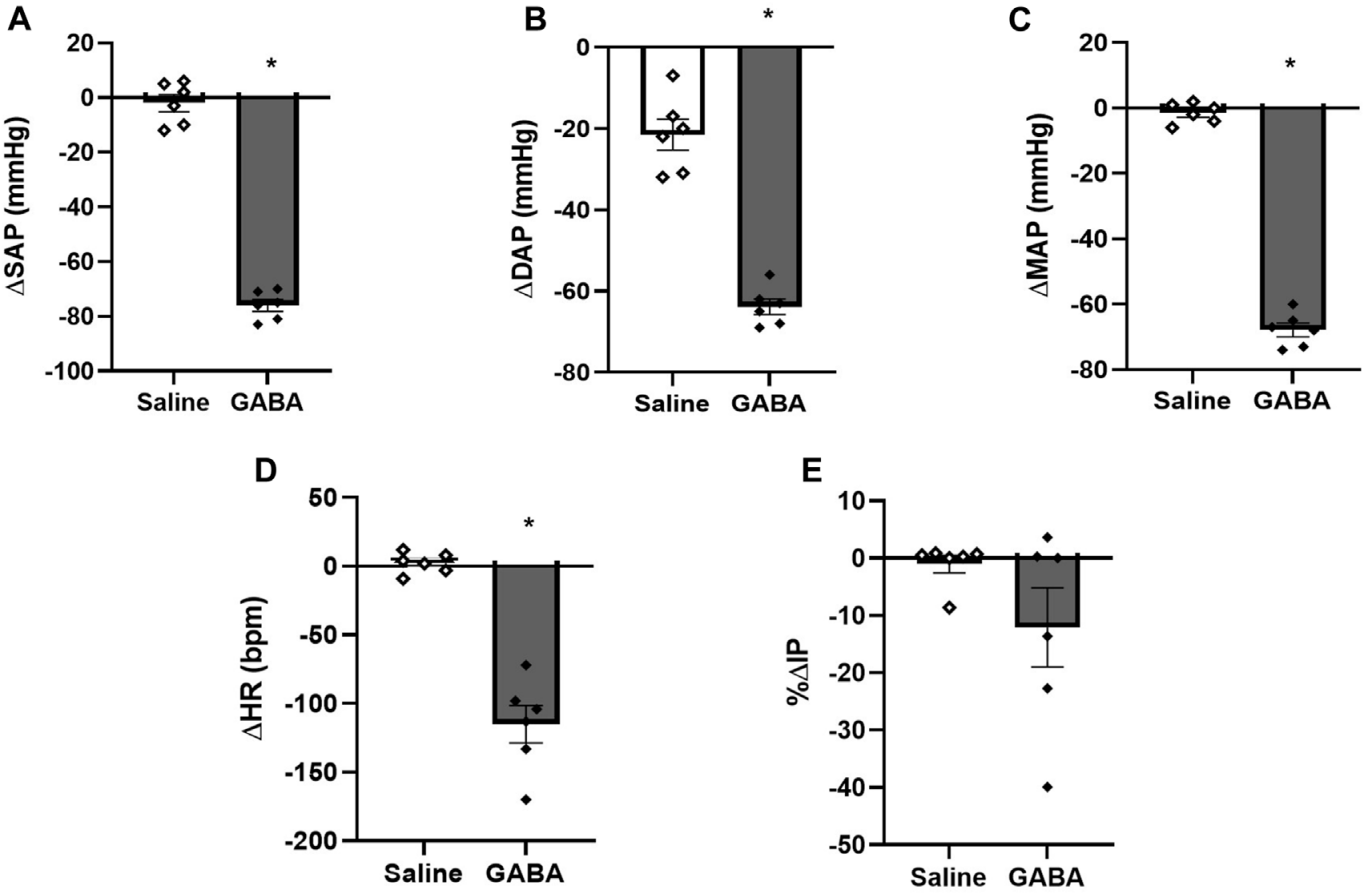
**(A)** Change in systolic arterial pressure (ΔSAP, mmHg), **(B)** diastolic arterial pressure (ΔDAP, mmHg), **(C)** mean arterial pressure (ΔMAP, mmHg), **(D)** change in heart rate (ΔHR, bpm), and **(E)** percent change in intravesical pressure (%ΔIP) induced by injection of saline (1 µL) or GABA (50 mM/μL, 1 µL) into the medial preoptic area (n = 6). *p < 0.05 vs. saline (paired Student’s t-test).

Injection of GABA (n = 6) into the mPOA evoked a significant fall in SAP (−76 ± 2 mmHg, *p* <0.0001), DAP (−64 ± 2 mmHg, *p* <0.0001), MAP (−68 ± 2 mmHg, *p* <0.0001) and HR (−115 ± 14 bpm, *p* = 0.0003) compared to saline injection (0 ± 3 mmHg, −1 ± 1 mmHg, −1 ± 1 mmHg and +4 ± 2 bpm, respectively, n = 6). However, no significant changes were observed in IP (−12.07% ± 6.92%) after GABA injection into the mPOA compared to saline injection (0.40% ± 015%) (Figures 3C; Figure 5). The latency for the peak responses in MAP and HR evoked by GABA injected in the mPOA was ∼8 min and the effect lasted for roughly 19 min.

### 5.3 Determination of gene expression of AT-1 receptor, ACE and GABAa receptor in the mPOA of rats (N = 6)

**FIGURE 6:**
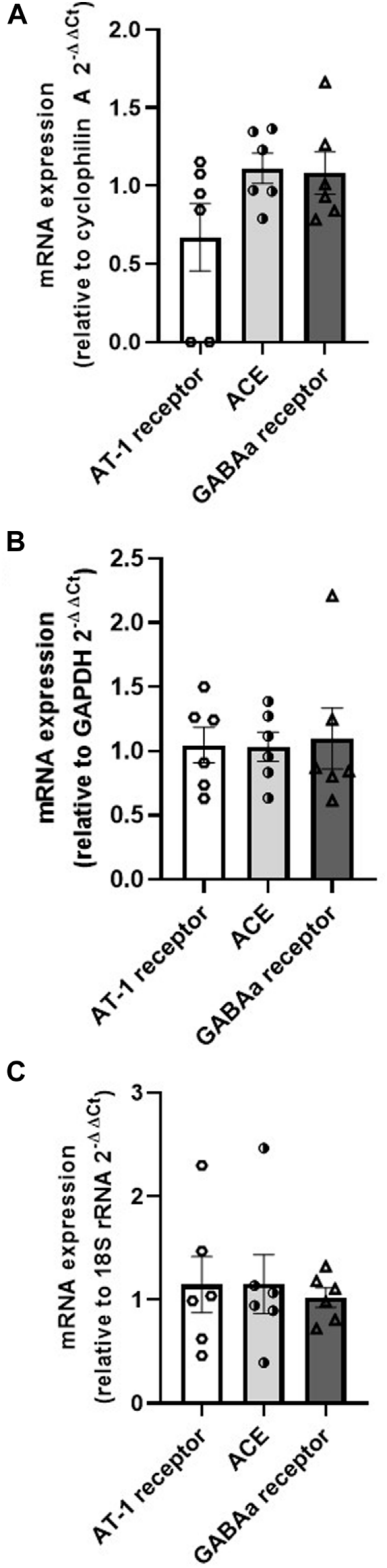
mRNA expression of AT-1 receptor, ACE and GABA in the medial preoptic area samples relative to the housekeeping genes **(A)** cyclophilin **(A,B)** GAPDH, and **(C)** 18S rRNA (N = 6).

The mRNA expression by RT-qPCR demonstrated that the AT-1 receptor (relative to cyclophilin A = 0.67 ± 0.22, relative to GAPDH = 1.05 ± 0.14, relative to 18S rRNA = 1.15 ± 0.27), ACE (relative to cyclophilin A = 1.11 ± 0.10, relative to GAPDH = 1.03 ± 0.11, relative to 18S rRNA = 1.15 ± 0.28), and GABAa receptor (relative to cyclophilin A = 1.08 ± 0.13, relative to GAPDH = 1.10 ± 0.24, relative to 18S rRNA = 1.02 ± 0.09) are found in the mPOA samples (n = 6) (Figure 6).

## 6 Discussion

The findings of this study in female rats demonstrate that Ang II injection into the mPOA yielded hypotension, bradycardia and increase in intravesical pressure. The blockade of AT-1 receptors in the mPOA by losartan elicited no significant changes in any variable studied, even though the dose administered was high in comparison to Ang II. Despite the GABAergic inhibition of mPOA evoked a marked hypotension and bradycardia similarly to Ang II, no change was observed in intravesical pressure. The present data also showed that the genes for AT-1 receptors, ACE and GABAa receptor were found in the mPOA. The mPOA samples for gene expression were obtained from intact brains, without guide cannula implantation in the mPOA, in order to exclude any influence of previous surgery on the integrity of mPOA neurons.

Although the volume of injection (1 µL) in the medial preoptic area could be considered large, the responses evoked by Ang II or GABA injections were only observed when the injection spot was centered in the medial preoptic area. Injections of Ang II or GABA located out of the medial preoptic area either more dorsal or more lateral from the midline, which was confirmed by the histological analysis, caused no change in the cardiovascular parameters and intravesical pressure. These findings suggest that the responses elicited by Ang II or GABA injections observed in the current study were specific and targeted to medial preoptic area.

The fact that the preoptic area is involved in the autonomic modulation has been known since 1935, when Kabat et al. (1935) demonstrated blood pressure changes to electrical stimulation of the POA in anesthetized cats. Nevertheless, Wang and Ranson. (1941) suggested that the preoptic area could play a dual effect on the autonomic nervous system, leading to vagal activation and causing sympathetic inhibition as the electrical stimulation elicited bradycardic response that was significantly attenuated by vagotomy. Folkow et al. (1959) have also shown that a pronounced hypotension and bradycardia can be evoked by electrical stimulation of the preoptic area in vagotomized cats with bilateral carotid occlusion, suggesting its sympathoinhibitory role. The electrical stimulation would be able to activate either cell bodies of neurons and axons, thereby those earlier studies causing nonspecific effects could also activate neurons lying outside the preoptic area. In 2017, Fassini et al. (2017) have inhibited the mPOA neurotransmission using cobalt chloride, a non-selective inhibitor of synapses. They demonstrated that this brain area exerts a tonic inhibitory function on cardiac sympathetic tone under resting and stress conditions, negatively modulating the sympathetic component of the baroreflex. However, the studies of could not demonstrate which neurotransmitter or neuromodulator would be released in the mPOA synapses to produce those effects.

In our study, either Ang II or GABA injections in the mPOA caused hypotensive and bradycardic responses, nevertheless, the latency to achieve the peak responses, intensity and duration of the responses were not equivalent. Further, the effects on intravesical pressure were different after Ang II and GABA injections in the mPOA. We observed an increase in intravesical pressure elicited by Ang II, whilst no change in intravesical pressure was caused after GABAergic inhibition. Hence, our data are suggestive that Ang II and GABA could be involved in the sympathoinibitory pathways to reduce blood pressure and heart rate, however are likely acting in different central pathways involved in micturition control.

Even though it is classically known that GABA injections induces responses with short latency and duration in areas involved in cardiovascular control, we observed a long latency for the appearance of the hypotensive response and bradycardia elicited by GABA injection in the mPOA. In addition, these responses showed a long duration. Despite we do not know the explanation for this fact, which is a limitation of this study, recent studies have also shown that injections of GABA into the shell Nucleus Accumbens evokes a huge hypotension and bradycardia, which are also long-lasting responses (∼14 min) (de Carvalho et al., 2023), that is quite unusual considering the classically known shorter responses evoked by GABA.

In the present study, the blockade of AT-1 receptors with losartan injection in the mPOA elicited no significant change on resting arterial pressure, heart rate and intravesical pressure. The absence of any noticeable responses evoked by losartan suggests that neurons with AT-1 receptors in the mPOA, where Ang II binds, exert a phasic control on the pathways which regulate these physiological parameters.

In the current study, we have not tested the Ang II after losartan injection in the mPOA, which is a limitation of this study. Nonetheless, qPCR demonstrated that AT-1 receptors, ACE and GABAa receptors mRNA are present in the mPOA. The mPOA contains the mRNA for angiotensinogen, low density of fibers with immunoreactivity for Ang II, modest immunoreactivity for AT-1 receptors and moderate amounts of ACE. In contrast, immunoreactivity for angiotensinogen has not been reported (Bunnemann et al., 1983). Thus, our data showing the existence of AT-1 receptors and ACE mRNA in the mPOA is consistent with the protein expression showed by Bunnemann et al. (1983). Taken together with the fact that Ang II and GABA gave similar responses to cardiovascular parameters, but distinctly on urological, it is evident that the agonists acted selectively on the different neural pathways.

Formation of Ang II is dependent on cleavage of Angiotensin I, which undergoes the action of ACE (Chappell et al., 2000; Rice et al., 2004). Since ACE mRNA was expressed in the mPOA, it is likely that neurons in the mPOA synthetizes Ang II, which is released in the synapses of this brain area to bind AT-1 receptors, in order to exert a phasic control of the sympathetic pathways involved in cardiovascular regulation. Despite we have not performed the gene expression of AT-2 receptors, ACE-2 or Mas receptors in the mPOA, we cannot exclude the possibility that Ang II can bind to AT-2 receptors or can be transformed to Angiotensin-(1–7) and bind to Mas receptors.

Studies by Bastos et al. (1994) have shown that Ang II injected in the mPOA in conscious male Holtzman rats induces thirst (dipsogenic effect), natriuresis, kaliuresis and diuresis. In addition, Ang II at the dose of 25 ng injected into the mPOA in conscious rats evoked a pressor response, which was attenuated by previous blockade of α1-adrenoceptors with prazosin, but not by antagonism of β1/β2 adrenoceptors with propranolol. In our study, we observed a depressor response (hypotension) after Ang II injection at the concentration of 0.1 nM (in a volume of 1 µL) in the mPOA in isoflurane anesthetized rats. Two different hypotheses could underpin the difference in the blood pressure responses. One of them could be the dose of Ang II, which was very low in our study (0.1046 pg), whilst in the study of Bastos et al. (1994), the dose injected in the mPOA was 25 ng (25,000 pg). Tentatively, a part of the differences in response could further be attributed to the anesthetic used in our study that could facilitate a depressor response.

Rusyniak et al. (2011) have shown that injection of muscimol, a GABAa receptor agonist, into the mPOA yielded a pressor response and tachycardia in conscious male Sprague-Dawley rats. Unlike the reports of Rusyniak et al. (2011), the present study showed that GABA injection in the mPOA in isoflurane-anesthetized rats caused a marked hypotensive response. The existence of GABAa receptor mRNA in the mPOA samples currently demonstrated suggests that GABA binds to these receptors in this brain area. However, it is noteworthy that the center of drug injection in the mPOA in the study of Rusyniak et al. (2011) was in a more rostral level compared to the present study. Hence, it is unknown if different populations of neurons and/or pathways in the mPOA could be affected by muscimol or GABA injections leading to opposite responses. Another source of difference to the findings of Rusyniak et al. (2011) could once again be the anesthesia used in the current study.

Micturition in neonatal rats is mediated by a spinal reflex pathway activated when the mother licks the perineum to produce an intense bladder contraction and urination. However, bladder distention, unlike what occurs in adult animals, does not induce reflex urination in neonates. As the central nervous system matures during the postnatal period, the spinal reflex is gradually replaced by a spinobulbospinal reflex pathway that is the primary mechanism for reflex urination in adult animals. This pathway has an integration center in the rostral pons and peripheral afferent and efferent pathways (Sugaya et al., 1997). Pseudorabies virus labels many neurons in various brain regions (PRV) after injected into the urinary bladder of rat pups. The distribution of PRV-infected neurons is somewhat broader than in adult mice. In adult rats, the mPOA shows labeled neuronal populations, although with less expression compared to other brain areas. The results of the current study indicate that Ang II injected into the mPOA in adult rats increased intravesical pressure in anesthetized rats, whereas the inhibition of mPOA neurons by GABA did not affect the intravesical pressure. Thereby, Ang II in the mPOA is involved in the control of the central micturition pathways, and conversely the GABAergic transmission in the mPOA does not participate in the regulation of these pathways.

The mPOA has dense bi-directional connections with the periaqueductal gray matter (PAG), which sends descending projections to the rostral ventrolateral medulla (RVML) (Rizvi et al., 1996; Murphy et al., 1999). The RVLM contains the presympathetic neurons involved in cardiovascular regulation (Colombari et al., 2001). The PAG has neurons involved in central micturition control (Fowler et al., 2008). Hence, these projections from the mPOA to RVLM and PAG could underpin the neural substrate for the responses evoked by Ang II and GABA in the current study.

Although we have used female rats, the estrous cycle was not evaluated in each animal, which is a limitation of this study. Nevertheless, the rats were maintained in groups of 4 animals per cage and their estrous cycles were likely synchronized (McClintock, 1978; McClintock, 1981). Our data was obtained in different days and over several months, with rats likely in different phases of the estrous cycle. Despite that, the changes in the cardiovascular parameters and intravesical pressure evoked by Ang II or GABA injections in the mPOA did not show huge variability, which suggests that the estrous cycle does not seem to influence the responses observed in the current study. We have not carried out the same studies in male rats. It is unknown whether the responses evaluated will be different or not depending on the gender, and this will require further investigation.

In conclusion, the Ang II mediated transmission in the mPOA has the capacity to affect the cardiovascular regulation causing hypotension and bradycardia, and further eliciting increase in intravesical pressure in anesthetized female rats, which also suggests an involvement in the control of central micturition pathways. In addition, a phasic control dependent on AT-1 receptors in the mPOA seems to be involved in the regulation of those cardiovascular and intravesical parameters. In contrast, GABAergic transmission in the mPOA participates in the pathways of cardiovascular control eliciting hypotension and bradycardia in anesthetized female rats, nevertheless, this neurotransmission is not involved in the micturition control.

## Data Availability

The original contributions presented in the study are included in the article/Supplementary Material, further inquiries can be directed to the corresponding author.
